# Complete Genome Sequence of *Campylobacter fetus* subsp. *venerealis* Biovar Intermedius, Isolated from the Prepuce of a Bull

**DOI:** 10.1128/genomeA.00526-13

**Published:** 2013-08-01

**Authors:** Gregorio Iraola, Ruben Pérez, Hugo Naya, Fernando Paolicchi, David Harris, Trevor D. Lawley, Natalia Rego, Martín Hernández, Lucía Calleros, Luis Carretto, Alejandra Velilla, Claudia Morsella, Alejandra Méndez, Andrea Gioffre

**Affiliations:** Sección Genética Evolutiva, Facultad de Ciencias, Montevideo, Uruguaya; Unidad de Bioinformática, Institut Pasteur de Montevideo, Montevideo, Uruguayb; Departamento de Producción Animal y Pasturas, Facultad de Agronomía, Universidad de la República, Montevideo, Uruguayc; Laboratorio de Bacteriología, Unidad Integrada INTA-Universidad Nacional de Mar del Plata, Balcarce, Argentinad; Wellcome Trust, Sanger Institute, Hinxton, Cambridgeshire, United Kingdome; Instituto de Biotecnología, Instituto Nacional de Tecnología Agropecuaria, Buenos Aires, Argentinaf

## Abstract

*Campylobacter fetus* subsp. *venerealis* is the causative agent of bovine genital campylobacteriosis, a sexually transmitted disease distributed worldwide. *Campylobacter fetus* subsp. *venerealis* biovar Intermedius strains differ in their biochemical behavior and are prevalent in some countries. We report the first genome sequence for this biovar, isolated from bull prepuce.

## GENOME ANNOUNCEMENT

*Campylobacter fetus* is an important veterinary pathogen. This species is currently divided into two subspecies, *Campylobacter fetus* subsp. *fetus*, causative of abortion in sheep, and *Campylobacter fetus* subsp. *venerealis*, the etiologic agent of bovine genital campylobacteriosis (1), a disease that has spread worldwide and causes economic losses mainly in countries where natural breeding is frequent (2). A distinct group of *C. fetus* strains known as *C. fetus* subsp. *venerealis* biovar Intermedius has also been determined; these strains phenotypically resemble *C. fetus* subsp. *venerealis*, but they react positively to the H_2_S test (typically positive for *C. fetus* subsp. *fetus*) (3). In recent years, an increase in the prevalence of this biovar has been noticed in some countries (e.g., South Africa) (4). However, the lack of genomic information for these atypical strains has hindered the development of molecular diagnostic tools and the study of their genomic evolution. Here we present the first complete genome sequence for *Campylobacter fetus* subsp. *venerealis* biovar Intermedius INTA 99/541, isolated from the prepuce of a naturally infected bull (5).

Sequencing was performed on an Illumina Hi-Seq 2000 platform and generated 13,953,630 paired-end reads (2 × 100 cycles). The resulting library was first corrected using ALLPATHS-LG (6) and then assembled with Velvet (7) software, producing 111 contigs with an average coverage of 130-fold. The assembly quality was improved using the PAGIT toolkit (8), based on the genome sequence of *C. fetus* subsp. *fetus* 82-40 (accession no. NC_008599) as the reference. The final assembly quality was evaluated with an assembly likelihood estimator (ALE) (9). The resulting pseudomolecule produced by contig scaffolding was automatically annotated with RAST (http://rast.nmpdr.org/).

*Campylobacter fetus* subsp. *venerealis* biovar Intermedius INTA 99/541 has a circular chromosome of 1,774,509 bp with an average GC content of 33%, including 2,421 putative protein-coding open reading frames (1.36 genes per kb), 3 rRNA operons, and 40 tRNA genes. BLAST analysis between those contigs that were not used in chromosome scaffolding and the GenBank plasmids database revealed high sequence homology with the pTet *Campylobacter jejuni* plasmid (accession no. NC_008790) and strong synteny conservation of Cpp protein-coding genes, important for plasmid mobilization (10). However, more extensive analyses are needed to confirm that this strain is carrier of extrachromosomal replicons.

Comparison between *C. fetus* subsp. *venerealis* biovar Intermedius INTA 99/541, *C. fetus* subsp. *venerealis* Azul-94 (10), *C. fetus* subsp. *venerealis* NTCT 10354^T^ (11), and *C. fetus* subsp. *fetus* 82-40 genomes using the Artemis comparison tool (12) revealed that sequence identity and synteny are conserved along genomes. Further analysis of these genomes will provide information regarding the basis of the unique physiological and biochemical features of *C. fetus* subsp. *venerealis* biovar Intermedius. Moreover, the availability of the first genome from this organism is an important achievement in the development of specific molecular tools for diagnosis and will shed light on the genomic evolution of *Campylobacter* species, although a representative number of genomes for this biovar will be needed to conduct more robust comparisons.

### Nucleotide sequence accession numbers. 

This whole-genome shotgun project has been deposited at DDBJ/EMBL/GenBank under the accession number ASTK00000000. The version described in this paper is version ASTK01000000.
